# Insulin-like growth factors and cancer: no role in screening. Evidence from the BUPA study and meta-analysis of prospective epidemiological studies

**DOI:** 10.1038/sj.bjc.6603200

**Published:** 2006-06-27

**Authors:** J K Morris, L M George, T Wu, N J Wald

**Affiliations:** 1 Centre for Environmental and Preventive Medicine, Wolfson Institute of Preventive Medicine, Barts and The London Queen Mary's School of Medicine and Dentistry, Charterhouse Square, London EC1M 6BQ, UK

**Keywords:** cancer screening, meta-analysis, cohort studies, insulin-like growth factors, epidemiology

## Abstract

Insulin-like growth factor-1 (IGF-1), insulin-like growth factor-2 (IGF-2), and insulin-like growth factor binding protein-3 (IGFBP-3) were measured in frozen serum samples from 1051 men with cancer and 3142 controls in a nested case–control study from the British United Provident Association (BUPA) study cohort and associations with 14 cancers were examined, including prostate, colorectal, and lung. A meta-analysis of studies on these three cancer sites was also conducted. In the meta-analysis the odds ratio between the highest quartile IGF-1 group and the lowest quartile group was 1.31 (95% confidence interval (CI): 1.03–1.67) for prostate, 1.37 (1.05–1.78) for colorectal and 1.02 (0.80–1.31) for lung cancer, and for IGF-2 it was 0.72 (0.36–1.44) for prostate and 1.95 (1.26–3.00) for colorectal cancer. Results from the BUPA study were consistent with the estimates from the other studies. There were no statistically significant associations with IGFBP-3 and any of the cancer sites considered. Our results suggest that IGF-1, IGF-2, and IGFBP-3 measurements have no value in cancer screening, although IGF-1 and IGF-2 may be of aetiological significance in relation to colorectal and prostate cancer.

Prospective studies have shown that higher circulating concentrations of insulin-like growth factor 1 (IGF-1), insulin-like growth factor 2 (IGF-2), and insulin-like growth factor binding protein-3 (IGFBP-3) are associated with an increased risk of prostate cancer, colorectal cancer, and premenopausal breast, and ovarian cancer (Lukanova *et al*, 2002; Renehan *et al*, 2004). Aetiological importance has been attributed to these associations, and it has been suggested that they may be of value in screening for these cancers.

We here report results on measurements of IGF-1, IGF-2, and IGFBP-3 from the British United Provident Association (BUPA) prospective study, based on 1051 new cases of cancer at 14 different cancer sites, and we combine the results on prostate, colorectal, and lung cancer with those from published studies in a meta-analysis.

## METHODS

The BUPA study is a prospective study of 21 520 professional men aged 35–64 years resident in Britain who attended the BUPA medical centre in London between 1975 and 1982 for a routine medical examination. Serum samples were stored at −40°C. The men were flagged at the National Health Service Central Register in Southport, permitting automatic notification of death (with the certified cause) and cancer incidence by the Office for National Statistics. Further information on the causes of death was obtained by writing to each certifying doctor. This analysis is based on a maximum follow-up of 15 years and is restricted to those cancers for which at least 10 cases occurred. In this period, there were 1059 men for whom we had a cancer notification. For each case, three controls (who were still alive and for whom we did not have a cancer notification) were selected; they were matched for age and duration of storage of the serum sample each to one year.

In 2003 the frozen serum samples were retrieved. The sample was insufficient for eight cases; these and the corresponding controls were excluded. The sample was also insufficient in 11 controls, leaving 1051 cases, 1040 cases with three controls and 11 cases with two controls, 3142 controls in total. IGF-1, IGF-2 and IGFBP-3 were measured using DSL-10-5600 ACTIVE IGF-I, DSL-10-9100 ACTIVE IGF-2 and DSL-10-6600 ACTIVE IGFBP-3 enzyme-linked ImmunoSorbent Assay (ELISA) kits. The serum samples from the cancer cases and their matched controls were systematically analysed together in the same analytical batch with blinding so that the case was not identifiable. The samples from the cases (and the corresponding controls) were analysed in groups according to the type of cancer.

To determine if freezing the samples and storing them at −40°C affected the levels of IGF-1 and IGF-2, a separate set of samples was analysed. Forty samples from the same BUPA cohort that the cases and controls came from (collected between 1975 and 1982) were randomly selected, five from each year of the study. Forty fresh samples were obtained from men aged 35–64 years resident in Britain who attended the BUPA medical centre in London in 2003. The median levels from the samples prior to 1982 compared with the median in 2003 were 1% (95% confidence interval (CI): −12% to +25%) higher for IGF-1 (*P*=0.66) and −6% (95% CI: −18% to +4%) lower for IGF-2 (*P*=0.93), indicating that storing the serum at −40°C for over 20 years did not reduce the levels of IGF-1 or IGF-2.

The statistical analysis of the BUPA data was carried out as follows: Pearson correlation coefficients were used to examine the associations between IGF-1, IGF-2, IGFBP-3 and age within the controls. The case–control matching was preserved for each cancer site, rather than analysing the cases of a particular cancer with all the controls. Conditional logistic regression models were used to calculate odds ratios (OR) for cancer for quartile levels of the markers, which takes account of the matching. Quartile cut points were determined on the distribution of control subjects for each cancer site separately. To determine if there was a significant linear association with risk of cancer the original markers (that is the continuous measurements not categorised into quartiles) were entered into the conditional logistic regression model. Body mass index (BMI), smoking and alcohol consumption were all investigated as possible confounders for each cancer site separately. BMI was analysed as a continuous measure. There were seven smoking categories (nonsmokers, exsmokers, pipe and cigar smokers and four categories of cigarette smokers (1–15 day^−1^, 15–24 day^−1^, 25–34 day^−1^ and 35+ day^−1^)) and five alcohol categories (Teetotal, ‘Social’, 1–2 units per day, 3–6 units per day and 6+ units per day).

The meta-analysis was carried out as follows: We searched MEDLINE and EMBASE from January 1996 to December 2005 combining the keywords IGF^*^ and cancer to find published prospective studies on IGF-1, IGF-2 and IGFBP-3 and cancer. We searched citation lists from review articles and previously published meta-analyses excluding breast and ovarian cancers, as our data are only on men. Meta-analyses were performed on the results using a random effects model for the published results from prospective studies (using a nested case–control design). The odds of cancer occurring in the top quartile of the marker compared with the bottom quartile were combined. For studies that gave the odds according to quintiles these were adjusted to quartile values. For prostate, colorectal and lung cancers we included the results of the present (BUPA) study in the meta-analysis. To assess for publication bias, we examined the funnel plots for each cancer site and tested if the regression of study estimate with study precision was significant. We investigated all sources of heterogeneity with subgroup analyses and also investigated the effects of adjusting IGF-1 for IGFBP-3 levels and IGFBP-3 for IGF-1 levels as is often perormed. Potential sources of heterogeneity investigated were: year of publication, type of sample, average time from sample collection to cancer diagnoses and study location. There was no evidence of publication bias for any of the cancer sites.

## RESULTS

Table 1 gives details of the study population including the number of men according to cancer site and the mean age of the men at the time of entry into the BUPA study and gives the medians and interquartile ranges for IGF-1, IGF-2 and IGFBP-3 according to case–control status and, as has been observed in other studies, IGF-1 and IGFBP-3 were moderately correlated (*r*=0.5) and both declined with age (*r*=−0.18 for IGF-1 and age and *r*=−0.12 for IGFBP-3 and age). IGF-2 was less highly correlated with IGFBP-3 (*r*=0.29) and age (*r*=−0.10).

### Prostate, colorectal and lung cancer results from the BUPA study

Table 2 shows the odds of cancer according to quartiles (fourths) of IGF-1, IGF-2 and IGFBP-3 for prostate, colorectal and lung cancer. There were no statistically significant associations between any of the cancers and any of the serum markers but the wide confidence did not exclude relative risks of up to 3.4.

### Results of the meta-analysis

**Figure 1:**
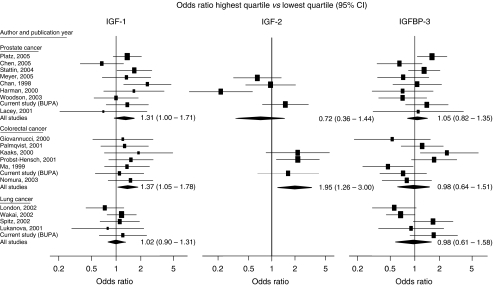
Prospective studies of IGF-1, IGF-2 and IGFBP-3 and prostate, colorectal and lung cancer. The studies are ordered (top down) by increasing average time interval between blood collection and cancer diagnosis.

Table 3 and Figure 1 shows the meta-analysis of IGF-1, IGF-2 and IGFBP-3 results from published prospective studies and the results from the present study (there were no published studies on IGF-2 and lung cancer). This analysis includes all the studies in the earlier analysis by Renehan *et al* (2004) together with eight others (Lacey *et al*, 2001; Wakai *et al*, 2002; Nomura *et al*, 2003; Woodson *et al*, 2003; Stattin *et al*, 2004; Chen *et al*, 2005; Meyer *et al*, 2005; Platz *et al*, 2005). The meta-analysis was restricted to data from prospective studies to minimise any effects of the cancer on the measurements made.

#### Prostate cancer

The risk of developing prostate cancer for men in the top quartile of IGF-1 compared with the bottom quartile was 1.31 (95%CI: 1.03–1.67; Table 3). There was no significant heterogeneity between studies (*P*=0.21). There was no association between the risk of prostate cancer and IGF-2 (OR=0.72; 95% CI: 0.36–1.44; Table 3) or IGFBP-3 levels (OR=1.05; 95%CI: 0.82–1.35; Table 3).

In the present study prostate-specific antigen (PSA) was also measured on the 141 men who developed prostate cancer and their controls at the same time as measuring IGF-1. The odds ratio of prostate cancer in the highest quartile compared with the lowest quartile was very high (OR=31 95% CI: 10–92), demonstrating by comparison the modest association with IGF-1, in spite of it being statistically significant.

#### Colorectal cancer

There was a positive association (OR=1.37 95% CI: 1.05–1.78) with IGF-1 and no evidence of heterogeneity between the studies (*P*=0.68). There was also a positive association (OR=1.95 95%CI: 1.26–3.00) with IGF-2 and again no evidence of heterogeneity between the studies (*P*=0.87). For IGFBP-3 there was no significant association (OR=0.98 (0.64–1.51), however, there was significant heterogeneity between studies (*P*=0.02). None of the subgroup analyses explained the heterogeneity between the studies.

#### Lung cancer

There was no indication of an association between IGF-1 and lung cancer (OR=1.02 (0.80–1.31)) nor between IGFBP-3 and lung cancer (OR=0.98 (0.61–1.58)). However, there was significant heterogeneity between the studies for IGFBP-3 (*P*=0.01). The study by Spitz *et al* (2002) differed from the other studies in that the cohort was of men who were heavy smokers and asbestos workers. Excluding this study from the analyses resulted in an odds ratio=0.84 (0.52–1.35) with a reduction in the heterogeneity (*P*=0.07) and there was evidence that the study differed significantly from all the other studies (*P*=0.002).

### BUPA study results on 11 other site-specific cancers

The results are presented according to tertile group (not quartile groups) because of the smaller numbers (Table 4). Cancers of the oesophagus, mouth and pharynx were significantly associated with lower levels of IGF-1 and cancer of the lymphoma was significantly associated with lower levels of IGF-2. There were no statistically significant associations between the 11 cancer sites with IGFBP-3. The three associations are likely to be spurious results due to multiple significance testing. There were no significant results when all cancers were considered together for any of the markers.

## DISCUSSION

Our results show that none of the growth factors measured (IGF1, IGF2 or IGFBP3) have any value in cancer screening in men. The strength of the associations are too weak for them to have any useful effect in distinguishing people who will and will not develop cancer at any of the sites studied. Relative risks of about two between the top and bottom quartile groups translate into a sensitivity (or detection rate) of less than 10% for a 95% specificity (5% false-positive rate) (Wald *et al*, 1999). In contrast, the corresponding odds ratio for prostate cancer between the top and bottom quartile groups of PSA measured in this study was 31, corresponding to a sensitivity of over 30% for a 95% specificity, high enough for a potential screening test (Wald *et al*, 1999). Combining the growth factors with other markers into a screening test is also unlikely to be worthwhile, as in order to obtain a reasonable screening performance over 20 tests with relative risks of about two would be required (Wald *et al*, 2005).

Results relating to three female cancers, breast, ovary and endometrium, were not included in our meta-analyses as they were discussed in a recent meta-analysis by Renehan *et al* (2004) (breast) and two papers by Lukanova *et al* (2002, 2004) (ovary and endometrium). Renehan *et al* (2004) reported a relative risk of premenopausal breast cancer between the top and bottom quartile of IGF-1 of 2.08 (1.37–3.15), with no significant associations for postmenopausal breast cancer. Lukanova *et al* (2002, 2004) reported a nonsignificant positive association between IGF-1and premenopausal ovarian cancer (RR=1.90 (0.63–5.75)) with no other associations approaching statistical significance. The strengths of the associations between these female cancers and the growth factors measured are not higher than any observed in our study. There are no data relating to cervix cancer, but given the overall pattern of results it would be surprising if cervix cancer was an exception. We therefore are confident that the general conclusion that none of the growth factors measured (IGF1, IGF2 or IGFBP3) have any value in cancer screening can be extended to all the main cancers in men and women.

Our second conclusion is that one of the growth factors, IGF2, may be of aetiological significance in colorectal cancer. This was suggested by the results from the two previous studies and is now clarified by the meta-analysis including our own results on this growth factor. Statistically significant associations between IGF-1 and colorectal and prostate cancer may also be of aetiological significance, but the evidence is weaker.

This is the first report of a cohort study on associations with cancers in men other than prostate, colorectal and lung. Considering the small numbers of cancers diagnosed and the likely OR of around 1.5, this study has low power to detect significant associations in the less common cancers, but nonetheless there were no positive associations between any of the cancers and any of the markers. The value of this study is to enable the data to be included in future meta-analyses needed to have the statistical power to investigate possible associations. We restricted the analysis of the BUPA data to 15 years of follow-up since in the other studies the meta-analysis had similar maximum lengths of follow up. The data were also available for a further seven years of follow-up, but their inclusion did not materially alter the results.

The published studies in the meta-analysis presented the odds for IGF-1 adjusted for IGFBP-3 values and the odds for IGFBP-3 adjusted for IGF-1. Our meta-analysis provides no evidence that IGFBP-3 is associated with cancer and therefore there is no reason to adjust IGF-1 values for IGFBP-3. Such an analysis yields similar estimates of the OR obtained for IGF-1 alone, confirming that it is of little or no value in increasing the strength of the association.

## Figures and Tables

**Table 1:** BUPA study: characteristics at the time of screening of men who developed cancer within 15 years and controls who did not

	**Controls**	**Cases**
Number of men	3142	1051
Mean age (years)	52.4	52.4
Median IGF-1 (ng ml^−1^) (25^th^-75th centiles)	124 (90–160)	122 (88–164)
Median IGF-2 (ng ml^−1^) (25th–75th centiles)	636 (517–767)	639 (514–772)
Median IGFBP-3 (*μ*g ml^−1^) (25th-75th centiles)	3.2 (2.7–3.8)	3.2 (2.6–3.8)
Median time till diagnosis in cases (years)		9.7
Proportion cases diagnosed within 3 years		8%
Proportion cases diagnosed within 10 years		53%
		
*Number of men by site of cancer*		
Prostate	423	141
Colon and rectum	440	147
Lung	498	167
Bladder	234	78
Lymphoma	166	56
Stomach	123	41
Oesophagus	123	41
Skin	123	41
Pancreas	114	38
Brain	113	38
Kidney	110	37
Leukaemia	102	34
Mouth and pharynx	39	13
Larynx	38	13
Other/Multicancer	496	166

**Table 2:** BUPA study: odds of prostate, colorectal, and lung cancer according to quartiles of serum levels of IGF-1, IGF-2 and IGFBP-3 within the first 15 years of follow-up (follow-up being the time between sample collection and diagnosis)

		**Adjusted odds ratios^a^**				
		**Quartile groups**				
**Site of cancer**	**Analyte**	**1 (ref)**	**2**	**3**	**4 (95% CI)**	***P* for trend**
*Prostate*						
	IGF-1	1	0.96	0.61	1.37 (0.76–2.49)	0.62
	IGF-2	1	1.18	1.49	1.47 (0.77–2.81)	0.19
	IBFBP-3	1	1.09	0.84	1.40 (0.77–2.55)	0.42
*Colorectal*						
	IGF-1	1	1.20	1.39	1.10 (0.56–2.18)	0.65
	IGF-2	1	1.70	1.84	1.59 (0.67–3.75)	0.40
	IBFBP-3	1	0.90	1.06	0.72 (0.37–1.37)	0.46
						
*Lung*						
	IGF-1	1	1.00	1.23	1.21 (0.62–2.35)	0.45
	IGF-2	1	1.21	1.22	0.82 (0.39–1.73)	0.61
	IBFBP-3	1	0.90	1.39	1.70 (0.87–3.30)	0.06

a The odds ratios were adjusted for age by matching and were also adjusted: smoking for lung cancer and smoking, alcohol and body mass index for colon and rectum.

**Table 3:** Meta-analysis: prospective studies of IGF-1, IGF-2 and IGFBP-3 and cancer of the prostate, colon and rectum and lung, ordered by mean time till diagnosis

**Study**	**No. of cases**	**No. of control**s	**Gender**	**Sample medium^a^**	**Adjust-ments^b^**	**Mean time till diagnosis (years)**	**Odds ratio highest quartile *vs* lowest quartile (95% CI)**		
							**IGF-1**	**IGF-2**	**IGFBP-3**
*Prostate cancer*									
Platz *et al* (2005)	462	462	m	Pl		2.2	1.37 (0.92–2.03)		1.62 (1.07–2.46)
Chen *et al* (2005)	174	175	m	Pl		3.4	0.67 (0.37–1.25)		0.65 (0.34–1.20)
Stattin *et al* (2004)	281	560	m	Pl	b,s	4.8	1.67 (1.02–2.71)		1.30 (0.84–2.03)
Meyer *et al* (2005)	100	400	m	Pl		6.8	1.34 (0.68–2.65)	0.67 (0.34–1.30)	0.72 (0.35–1.48)
Chan *et al* (1998)	152	152	m	Pl	s	7	2.41 (1.25–4.74)	0.97 (0.48–1.95)	1.07 (0.54–2.11)
Harman *et al* (2000)	72	127	m	Ser		9.2	1.65 (0.71–3.86)	0.24 (0.10–0.59)	0.71 (0.30–1.66)
Woodson *et al* (2003)	100	400	m	Ser	b	9.6	1.00 (0.54–1.87)		0.71 (0.36–1.39)
Current study (BUPA)	141	423	m	Ser		10.4	1.37 (0.76–2.49)	1.47 (0.77–2.81)	1.40 (0.77–2.55)
Lacey *et al* (2001)	30	60	m	Ser		14	0.70 (0.2–2.3)		1.1 (0.3–3.8)
									
Total	1512	2759				5.4	1.31 (1.03–1.67)	0.72 (0.36–1.44)	1.05 (0.82–1.35)
							Test for heterogeneity: *P*=0. 21	Test for heterogeneity: *P*=0. 01	Test for heterogeneity: *P*=0. 19
									
*Colorectal cancer*									
Giovannucci *et al* (2000)	79	158	F	Pl	a,b	3^c^	1.21 (0.52–2.81)		0.53 (0.18–1.53)
Palmqvist *et al* (2002)	168	336	M&f	Pl	s,l	3.35	1.27 (0.65–2.47)		1.23 (0.68–2.22)
Kaaks *et al* (2000); Hunt *et al* (2002)	102	200	F	Ser	s,l	4.8	1.88 (0.72–4.91)	2.08 (0.85–5.06)	2.46 (1.09–5.57)
Probst-Hensch *et al* (2001)	135	661	M	Ser	a,b,s	6.1	1.52 (0.82–2.85)	2.09 (1.14–3.82)	1.72 (0.91–3.25)
Ma *et al* (1999)	193	318	M	Pl	a,b,s	9^c^	1.36 (0.72–2.55)		0.47 (0.23–0.95)
Current study (BUPA)	147	440	M	Ser	a,b,s	9.6	1.10 (0.56–2.18)	1.59 (0.67–3.76)	0.72 (0.37–1.37)
Nomura *et al* (2003)	282	282	M	Ser	a,b,s	11	1.50 (0.8–2.8)		0.80 (0.4–1.6)
									
Total	1106	2395				7.6	1.37 (1.05–1.78)	1.95 (1.26–3.00)	0.98 (0.64–1.51)
							Test for heterogeneity: *P*=0.68	Test for heterogeneity: *P*=0.87	Test for heterogeneity: *P*=0.02
									
*Lung cancer*									
London *et al* (2002)	230	740	M	Ser	s	4	0.73 (0.43–1.24)		0.56 (0.30–1.03)
Wakai *et al* (2002)	194	9351	M&f	Ser	s,b	5.2	1.17 (0.78–1.77)		0.67 (0.45–1.01)
Spitz *et al* (2002)	159	297	M&f	Ser	s	6^c^	1.11 (0.64–1.93)		1.67 (0.96–2.92)
Lukanova *et al* (2001)	93	186	F	Ser	b,s,l	6.4	0.79 (0.29–2.19)		0.90 (0.36–2.25)
Current study (BUPA)	167	498	M	Ser	s	9.0	1.21 (0.62–2.35)		1.70 (0.87–3.30)
									
Total	843	11072				5.9	1.02 (0.80–1.31)		0.98 (0.61–1.58)
							Test for heterogeneity: *P*=0.64		Test for heterogeneity: *P*=0.01

a Pl=plasma, Ser=serum.

b All studies matched for age of cases. Other variables matched for are: a (alcohol consumption); b (body mass index); s (cigarette smoking); l (time since last food consumption); g(insulin and glucose).

c Mean time till diagnosis estimated from the papers.

**Table 4:** BUPA study: odds of cancer by tertile group of IGF-1, IGF-2 and IGFBP-3 for 11 common sites occurring during the first 15 years of follow-up (follow-up being the time between sample collection and diagnosis)

						**Odds ratios in tertile groups^a^**						
		**IGF-1**				**IGF-2**				**IGFBP-3**		
**Site of cancer**	**1**	**2**	**3 (95% CI)**	***P* for trend**	**1**	**2**	**3 (95% CI)**	***P* for trend**	**1**	**2**	**3 (95% CI)**	***P* for trend**
Bladder	1	1.09	1.09 (0.50–2.35)	0.83	1	0.79	0.90 (0.42–1.91)	0.73	1	1.22	1.08 (0.54–2.18)	0.83
Lymphoma	1	0.53	1.30 (0.59–2.86)	0.48	1	0.43	**0.42** (**0.18–0.99)**	**0.04**	1	0.74	0.58 (0.24–1.42)	0.23
Stomach	1	0.64	0.73 (0.28–1.94)	0.50	1	1.07	2.06 (0.51–8.41)	0.30	1	1.17	0.99 (0.37–2.68)	0.97
Oesophagus	1	0.59	0.21 (0.07–0.66)	**0.01**	1	0.57	1.20 (0.39–3.74)	0.48	1	0.87	1.34 (0.47–3.79)	0.60
Skin	1	1.11	2.26 (0.84–6.12)	0.10	1	2.05	2.27 (0.67–7.62)	0.15	1	2.09	1.90 (0.69–5.25)	0.22
Pancreas	1	0.50	0.68 (0.23–1.99)	0.48	1	0.63	0.48 (0.14–1.68)	0.24	1	0.62	0.74 (0.26–2.12)	0.59
Brain	1	1.13	1.18 (0.40–3.47)	0.77	1	0.56	1.06 (0.41–2.77)	0.90	1	0.48	0.44 (0.15–1.31)	0.12
Kidney	1	1.65	2.45 (0.85–7.07)	0.09	1	0.59	0.97 (0.26–3.62)	0.91	1	1.55	1.89 (0.66–5.48)	0.24
Leukaemia	1	0.57	1.20 (0.45–3.23)	0.75	1	1.02	0.50 (0.15–1.66)	0.25	1	1.18	0.61 (0.20–1.92)	0.37
Mouth and pharynx	1	0.25	0.10 (0.01–1.14)	**0.05**	1	1.36	1.20 (0.23–6.30)	0.83	1	0.24	0.33 (0.05–2.14)	0.19
Larynx^b^	1	0.23	1.09 (0.16–7.40)	0.92	—	—	—	—	1	19.99	5.33 (0.09–315.99)	0.82
Other/Multicancer	1	0.84	0.89 (0.54–1.46)	0.65	1	1.00	0.86 (0.50–1.49)	0.61	1	0.80	0.89 (0.55-1.44)	0.64
												
Total (incl prostate, colorectal and lung)	1	0.90	1.03 (0.84–1.27)	0.77	1	0.90	0.91 (0.71–1.15)	0.44	1	0.94	1.02 (0.83–1.25)	0.87

a The odds ratios were adjusted for age by matching and were also adjusted for the following confounding factors in the statistical analysis according to site : smoking (pancreas, larynx, mouth), alcohol (mouth, oesophagus) and body mass index (oesophagus, stomach).

b The odds ratios could not be calculated for IGF-2 and cancer of the larynx due to the lack of heterogeneity of IGF-2 values amongst cases and controls.
